# Circulating skeletal muscle related microRNAs profile in Piedmontese cattle during different age

**DOI:** 10.1038/s41598-021-95137-w

**Published:** 2021-08-04

**Authors:** Rupal S. Tewari, Ugo Ala, Paolo Accornero, Mario Baratta, Silvia Miretti

**Affiliations:** Department of Veterinary Sciences, University of Turin, Turin, Italy

**Keywords:** Genetics, Gene expression, Gene regulation, Physiology

## Abstract

Piedmontese cattle is known for double-muscle phenotype. MicroRNAs (miRNAs) play important role as regulators in skeletal muscle physiological processes, and we hypothesize that plasma miRNAs expression profiles could be affected by skeletal muscle growth status related to age. Plasma samples of cattle were collected during four different ages from first week of life until the time of commercial end of the fattening period before slaughter. Small-RNA sequencing data analysis revealed the presence of 40% of muscle-related miRNAs among the top 25 highly expressed miRNAs and, 19 miRNAs showed differential expression too. Using qRT-PCR, we validated in a larger bovine population, miRNAs involved in skeletal muscle physiology pathways. Comparing new-born with the other age groups, miR-10b, miR-126-5p, miR-143 and miR-146b were significantly up-regulated, whereas miR-21-5p, miR-221, miR-223 and miR-30b-5p were significantly down-regulated. High expression levels of miR-23a in all the groups were found. Myostatin, a negative regulator of skeletal muscle hypertrophy, was predicted as the target gene for miR-23a and miR-126-5p and we demonstrated their direct binding. Correlation analysis revealed association between miRNAs expression profiles and animals’ weights along the age. Circulating miRNAs could be promising for future studies on their biomarker potentialities to beef cattle selection.

## Introduction

Muscular phenotype is greatly influenced by several physiological changes underlying skeletal muscle growth and differentiation. Piedmontese breed is characterized by ‘double-muscle’ phenotype^1^ and for this reason has been subjected to intensive genetic selection to gain mighty yields of high-quality meat. Skeletal muscle hypertrophy is a complex process comprising of stimulation of quiescent skeletal muscle satellite cells and the proliferation and differentiation of myoblasts that transform into multinucleated myotubes^2,3^. In skeletal muscle tissue, several regulatory factors including microRNAs are involved in these dynamic processes^4,5^. MicroRNAs (miRNAs) are small non-coding RNAs, highly conserved among species, classified as negative modulators of gene expression at post-transcription level^6^. Indeed, miRNAs trigger either loss of function or degradation of target mRNA through establishing bond with 3′UTR^6^. Recent literature has shown several miRNAs to be involved in the regulation of myogenesis and differentiation of skeletal muscle tissue in a multitude of species^7–9^ miRNAs expressed exclusively in muscle have been named myomiRs^10^ and among these, miR-1, miR-133a/b and miR-206 have also been investigated in cattle^11,12^. An increasing number of studies have also identified other miRNAs involved in the post-natal skeletal muscle physiology and shed light on their mechanisms^13,14^. Particularly, miR-27b^15^, miR-26a^16^, miR-23a^17^, miR-143^18^, miR-126-5p^19^, and miR-146b^20^ have emerged as regulators during skeletal muscle cell proliferation, differentiation and hypertrophy. Interestingly, increasing evidence reveals that miRNAs can also be released from cells through active secretion or passively via membrane leaking and circulate in the bloodstream or other body fluids in a stable cell-free form^21^. Through this mechanism, circulating miRNAs (ci-miRNAs) can orchestrate cell-to-cell communication to regulate physiological and pathological pathways in recipient cells without being close to their origin^22^.

Up to now, in livestock, few studies about the expression and the role of specific miRNAs in body fluids are present but increasing attention has been due to the possibility to measure and take advantage of these molecules as biomarkers for tissue functions, production traits and early diagnosis of animals’ physiological state or disease^23,24^. To the best of authors’ knowledge, no information about plasma miRNAs expression pattern in beef cattle during different growing stages is available. Our study aimed to describe the expression of plasma miRNAs profiles previously identified as related with muscle tissue and investigated if they may be influenced according to age or body weight in Piedmontese cattle.

Specifically, this study focused on (1) to explore the panel of expression of ci-miRNAs in the plasma samples collected from Piedmontese cattle just after birth i.e., within the first week of life and during skeletal muscle growth until getting slaughtered at 15–17 months of age; (2) to identify which of these ci-miRNAs have gene targets involved in skeletal muscle hypertrophy; (3) to investigate about the possible relationship between muscle-related ci-miRNAs and body weight.

## Experimental design

**Figure 1:**
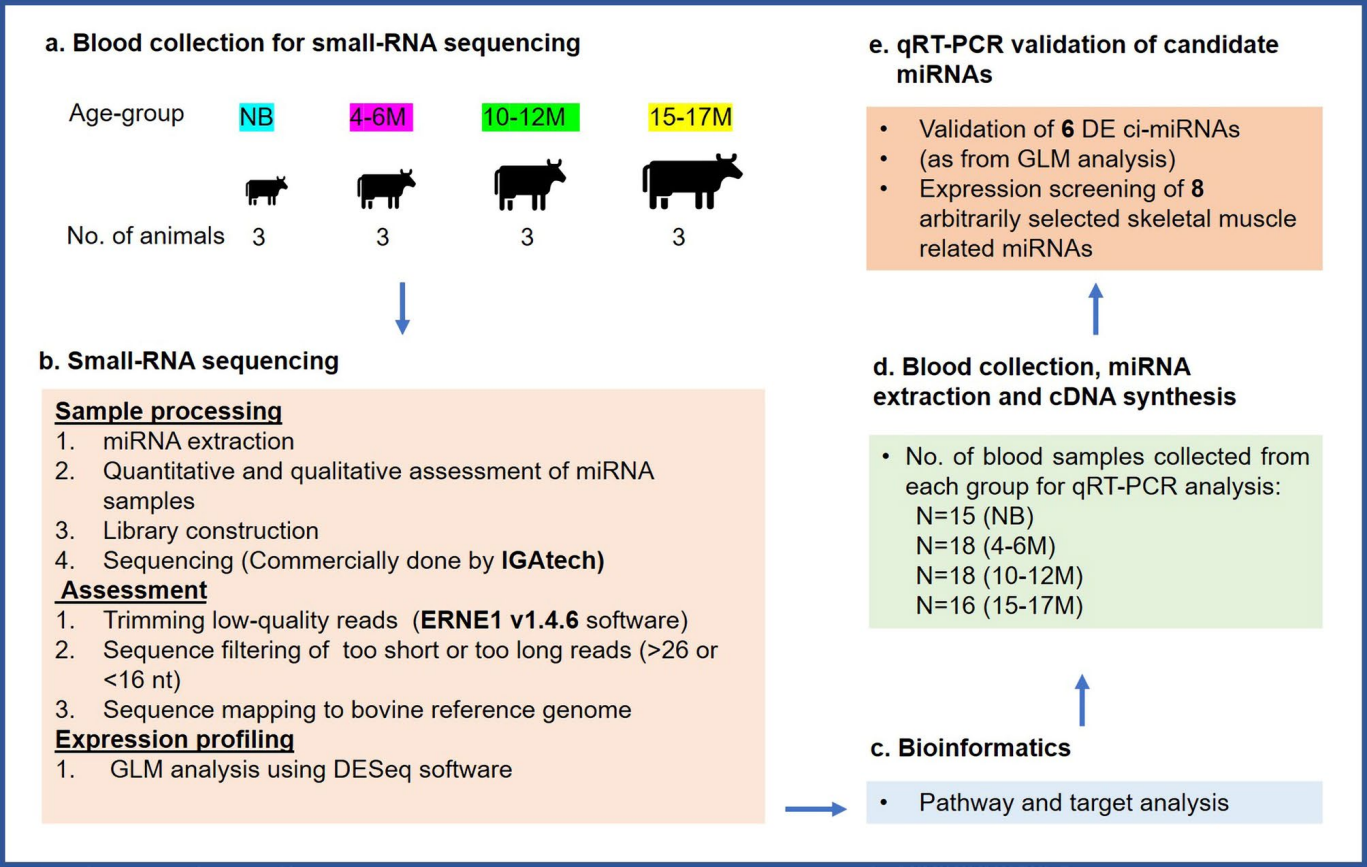
Schematic diagram of the study and workflow. (**a**) Target animal population divided into 4 groups (NB, 4–6M, 10–12M and 15–17M). Blood sampling was carried out from total 12 animals for Small RNA-Sequencing (3 per group). (**b**) For small RNA-Seq, sample processing (1 and 2) was performed whereas the sample processing (3 and 4), assessment (1, 2, 3) and expression profiling (1) were commercially performed by IGAtech, Italy. (**c**) Bioinformatic tools: TargetScan database was used to collect miRNA-mRNA target predictions to perform KEGG pathway analysis and specific target inspection. (**d**) Blood collection from total 67 animals, miRNAs extraction and cDNA synthesis. (**e**) qRT-PCR expression analysis of 14 selected miRNAs.

A pilot study to determine the presence and, the differential expression of bovine plasma miRNAs was strategized as depicted in Fig. 1. In brief, the target animals were divided into four age groups: new-born (NB), 4–6 months old (4–6M), 10–12 months old (10–12M) and 15–17 months old (15–17M) corresponding to different phases of skeletal muscle growth. Plasma miRNAs of three animals for each age group were sequenced to identify a panel of detectable and/or differentially expressed (DE-miRNAs) ci-miRNAs at four time points (Fig. 1a–c). The expression of candidate miRNAs using quantitative real-time PCR (qRT-PCR) was conducted using a larger bovine population, N = 15, N = 18, N = 18, and N = 16 animals of each age group, respectively (Fig. 1d,e). Body weight in kilograms during the four age periods beginning from birth until getting slaughtered was collected.

## Results

### miRNAs sequencing data

Plasma miRNA were sequenced to explore the panel of expression of ci-miRNAs in Piedmontese cattle across the four ages of growth and to identify DE-miRNA among the different groups. In total, 237 *Bos taurus* annotated miRNAs (bta-miRNAs) were detected. The number of reads per million (RPM) obtained for each sample after removing reads with low quality (reads without adapter, short reads and reads with multiple undetermined base calls are provided in Supplementary Table S1). Whereas Supplementary Fig. S1 displays the size distribution plot and read length distributions that showed a peak at 20–23 nucleotides, which is the main feature of mature miRNAs.

### Circulating miRNAs profile in four age-groups of beef cattle

The number of miRNAs with normalized expression between 0 and 100 RPM were 180, 181, 179 and 182 accounting for 75.9%, 75.9%, 75.5% and 76.7% respectively in NB, 4–6M, 10–12M and 15–17M groups. Likewise, the number of miRNAs with normalized expression between 100 and 1000 RPM were 73, 70, 68 and 67 during the four age periods tested, accounting for 23.6%, 24.7%, 26.3% and 33.1% of the total mature miRNAs, respectively. There were 30, 32, 31 and 35 miRNAs with mean expression above 1000, accounting for 9.7%, 11%, 11.9% and 17.3% of the total mature miRNAs, respectively. Lastly, the number of miRNAs with mean expression over 10,000 RPM at four stages were 4, 5, 4 and 6 respectively for each group, accounting for 2.7%, 2.9%, 3.1% and 4.1%, respectively (see Supplementary Fig. S2).

Out of 100 most expressed genes in blood plasma samples, the list of top 25 expressed genes was reported in Table 1. Among these, 10 (40%) were muscle-related miRNAs, including miR-486^25^, miR-26a^16^, miR-27b^15^, miR-30e-5p^26^, miR-30a-5p^27^, miR-146b^20^, miR-21-5p^28^, miR-660^29^, miR-186^30^, miR-126-5p^31^. Our previous study showed that skeletal muscle-specific miRNAs, namely miR-1, and miR-206 were abundant in the muscle tissues of Piedmontese cattle^32^, whereas they were detected in the plasma profiles with mean expression level less than 20 RPM, hence they were excluded for subsequent analysis^23^.

**Table 1:** The 25 most expressed miRNAs in blood plasma samples.

S. no.	miRNA
1	miR-486
2	miR-191
3	miR-148a
4	miR-423-5p
5	miR-26a
6	miR-let-7i
7	miR-25
8	miR-27b
9	miR-30d
10	miR-let-7f
11	miR-92a
12	miR-151-3p
13	miR-30e-5p
14	miR-30a-5p
15	miR-146b
16	miR-let-7a-5p
17	miR-21-5p
18	miR-660
19	miR-186
20	miR-6529a
21	miR-26b
22	miR-181a
23	miR-215
24	miR-let7c
25	miR-126-5p

According to the GLM analysis of sequencing data, 19 ci-miRNAs resulted differentially expressed (DE) (Table 2): 7 ci-miRNAs were downregulated and 12 ci-miRNAs upregulated.

**Table 2:** 19 miRNAs up- and downregulated according to GLM analysis of sequencing data.

S. no.	miRNAs	p value
1	miR-let-7e	0.03
2	miR-99b	0.01
**3**	**miR-126-5p**	0.0003
4	miR-10a	0.03
**5**	miR-1468	0.01
**6**	**miR-10b**	0.01
**7**	**miR-30a-5p**	0.0009
8	miR-15b	0.003
9	miR-342	0.01
10	**miR-143**	0.00006
11	**miR-99a-5p**	0.00006
12	miR-6119-5p	0.04
13	miR-374b	0.02
14	miR-150	0.003
15	miR-1296	0.0004
16	miR-1306	0.04
17	**miR-223**	0.02
18	miR-19b	0.03
19	miR-197	0.0003

### Pathway enrichment analysis (KEGG) of DE-miRNAs

**Figure 2:**
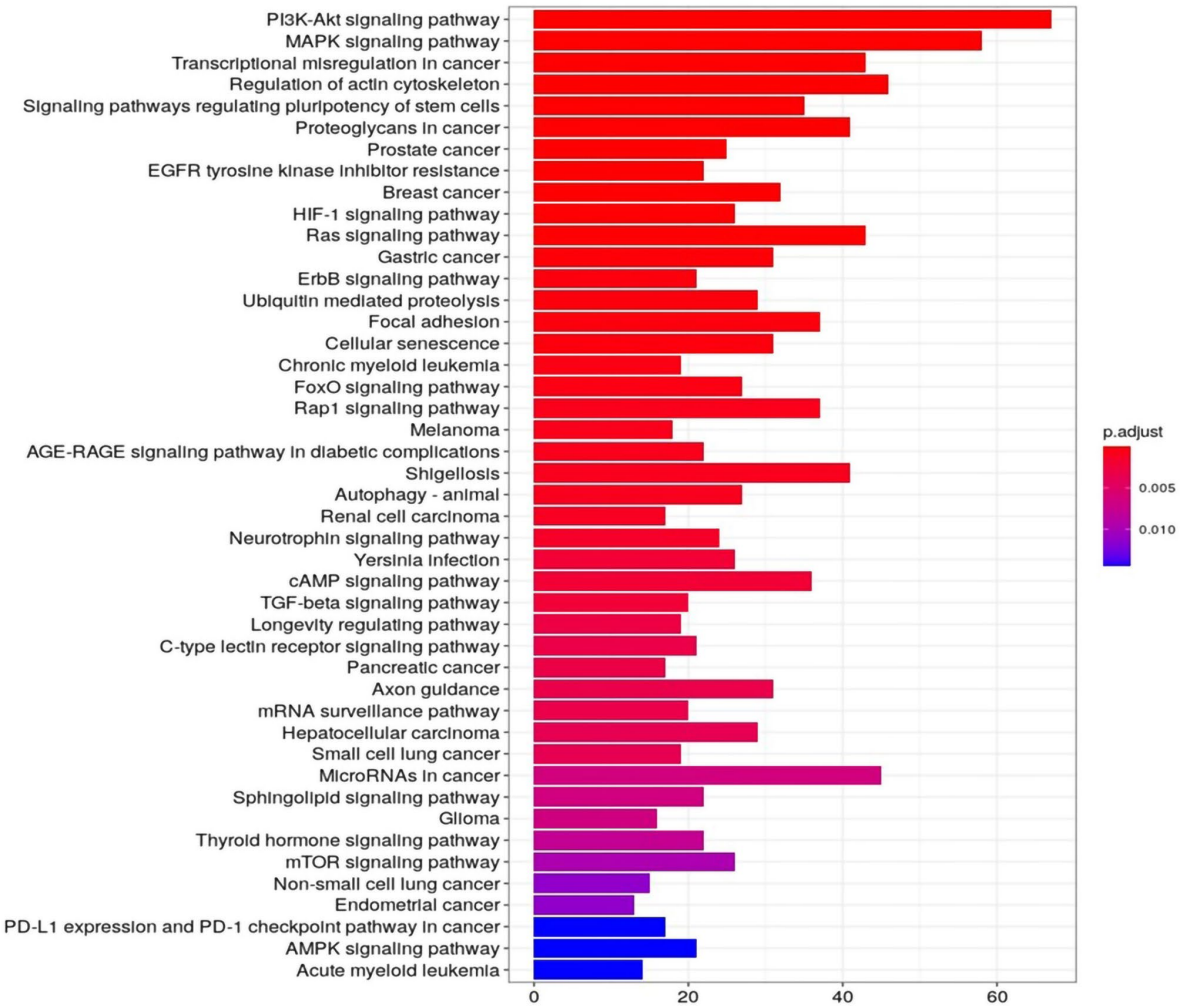
First 45 most significantly enriched KEGG pathways. On the X-axis, number of involved genes is reported, and the different colours (from red to blue) highlight different magnitude of the p value. The significantly enriched pathways were mainly involved in energy metabolism including PI3K-Akt and MAPK or regulating pluripotency of stem cells and skeletal muscle growth signalling pathways, including FoxO, TGF-β and mTOR signalling.

Starting from the DE-miRNAs, we identified putative target transcripts from TargetScan 7.2 database (as in the M&M section). These genes were then used to predict which specific molecular pathways showed possible participation in our biological setting. KEGG analysis showed statistically significant 65 pathways (Fig. 2). The significantly enriched pathways were mainly involved in energy metabolism including phosphatidylinositol 3-kinase (PI3K)/protein kinase B (AKT) signaling, signaling pathways regulating pluripotency of stem cells and skeletal muscle growth, including FoxO, transforming growth factor (TGF)-β and mTOR pathways.

### Age affects plasma miRNAs expression

To verify if age can affect the expression of selected ci-miRNAs, we decided to assay the skeletal muscle related miRNAs in a bigger population of bovines by qRT-PCR. Respectively 6 ci-miRNAs (miR-99a-5p, miR-143, miR-126-5p, miR-30a-5p, miR10b and miR-223) among the sequenced DE-miRNAs were selected for validation, and 8 miRNAs (miR-146b, miR-21-5p, miR-221, miR-30b-5p, miR23a, miR-155-5p, miR-660 and miR-30c-5p) were arbitrarily chosen from the complete list of sequenced miRNAs combining literature knowledge, number of reads (> 20 RPM), target genes prediction (TargetScan version 7.2) and KEGG pathway analysis.

**Figure 3:**
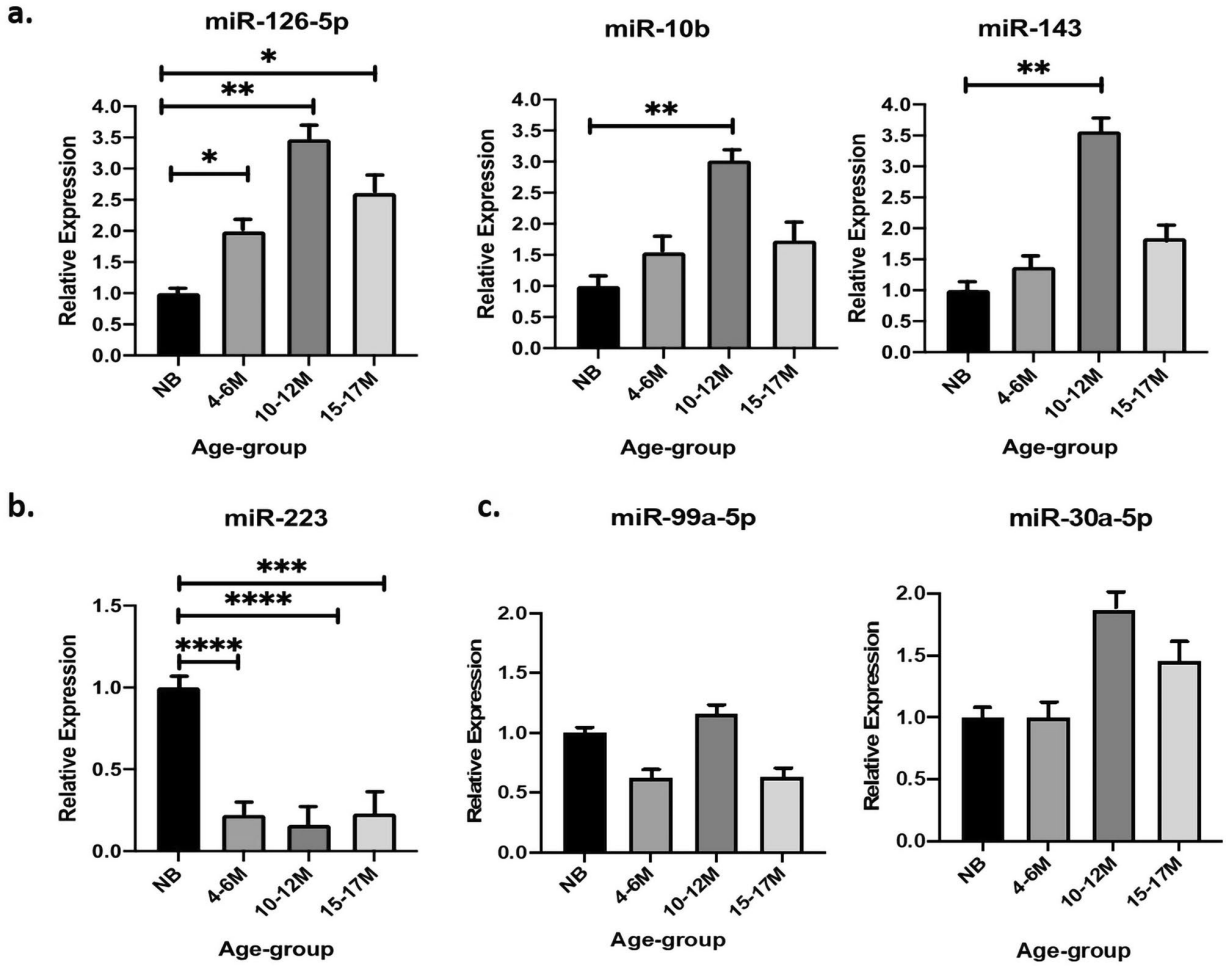
qRT-PCR validation of DE-miRNAs in sequencing results. Plasma miRNAs have been shown as DE in (**a**) upward direction (**b**) downward direction and (**c**) not DE. All expression levels were normalised to miR-378. Relative expression is shown as fold change (mean ± SEM) and statistical significance as *p < 0.05, **p < 0.005, ***p < 0.0005.

The relative abundance of all selected ci-miRNAs was quantified using miR-378 (stable number of reads and Cq values among the four groups) for normalization. The selected ci-miRNAs were detected in all the samples. When compared with NB, effect of the growing age was statistically significant for miR-10b, miR-143, miR-126-5p, miR-223 (Fig. 3a,b) while miR-99a-5p and miR-30a-5p, in contrast with sequencing results, have not shown significant variations in expression (Fig. 3c). miR-10b, miR-143, and miR-126-5p showed a rise in the expression during growth and reached the peak at 4–6 and 10–12 months of age (Fig. 3a); whereas the expression of miR-223 remained significantly decreased during the three growth periods (Fig. 3b).

**Figure 4:**
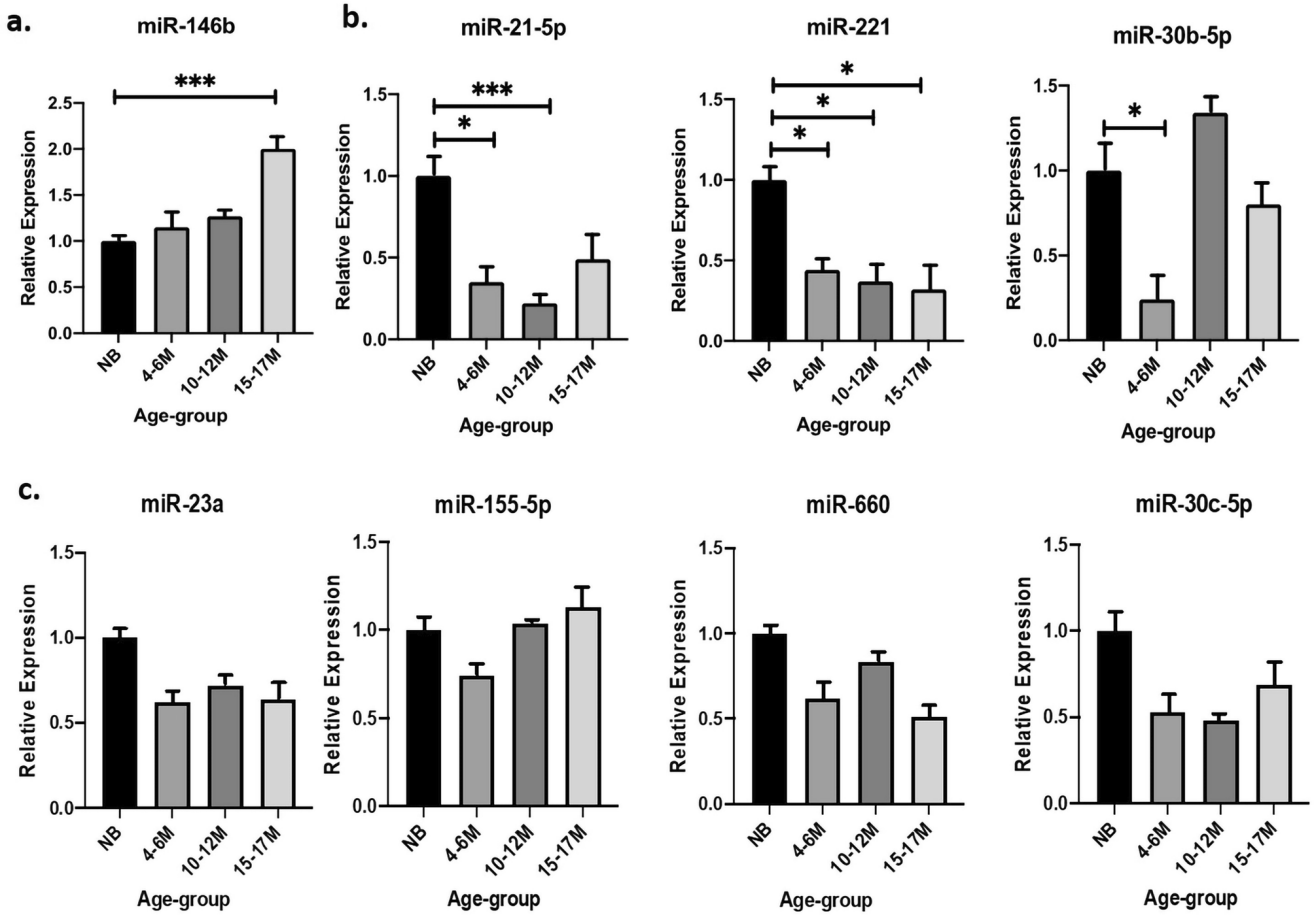
qRT-PCR expression profiles of arbitrarily selected skeletal muscle related miRNAs. Plasma miRNAs have been shown as DE in (**a**) upward direction, (**b**) downward direction and (**c**) not DE. Relative expression is expressed as fold change (mean ± SEM) and statistical significance as *p < 0.05, **p < 0.005, ***p < 0.0005.

Expression assay analysis of 8 arbitrarily selected ci-miRNAs was performed, and qRT-PCR revealed that miR-146b-5p expression increased in 15–17M old animals when compared with NB (Fig. 4a), whereas the expression levels of miR-21-5p, miR-221, miR-30b-5p decreased in growing animals (Fig. 4b). miR-23a, miR-155-5p, miR-660, and miR-30c-5p showed no significant differential expression among the groups (Fig. 4c). In view of the expression analysis results, we decided to investigate on putative target genes for ci-miRNAs that revealed high expression in terms of Cq values along the different age points (miR-23a) and ci-miRNAs with significant differential expression between NB and 10–12M groups, when the major peak of skeletal muscle increase was phenotypically evident (miR-10b, miR-126-5p, miR-143, miR-21-5p, miR-221 and miR-223).

### miR-23a and miR-126-5p directly target the 3′-UTR of bovine MSTN

**Figure 5:**
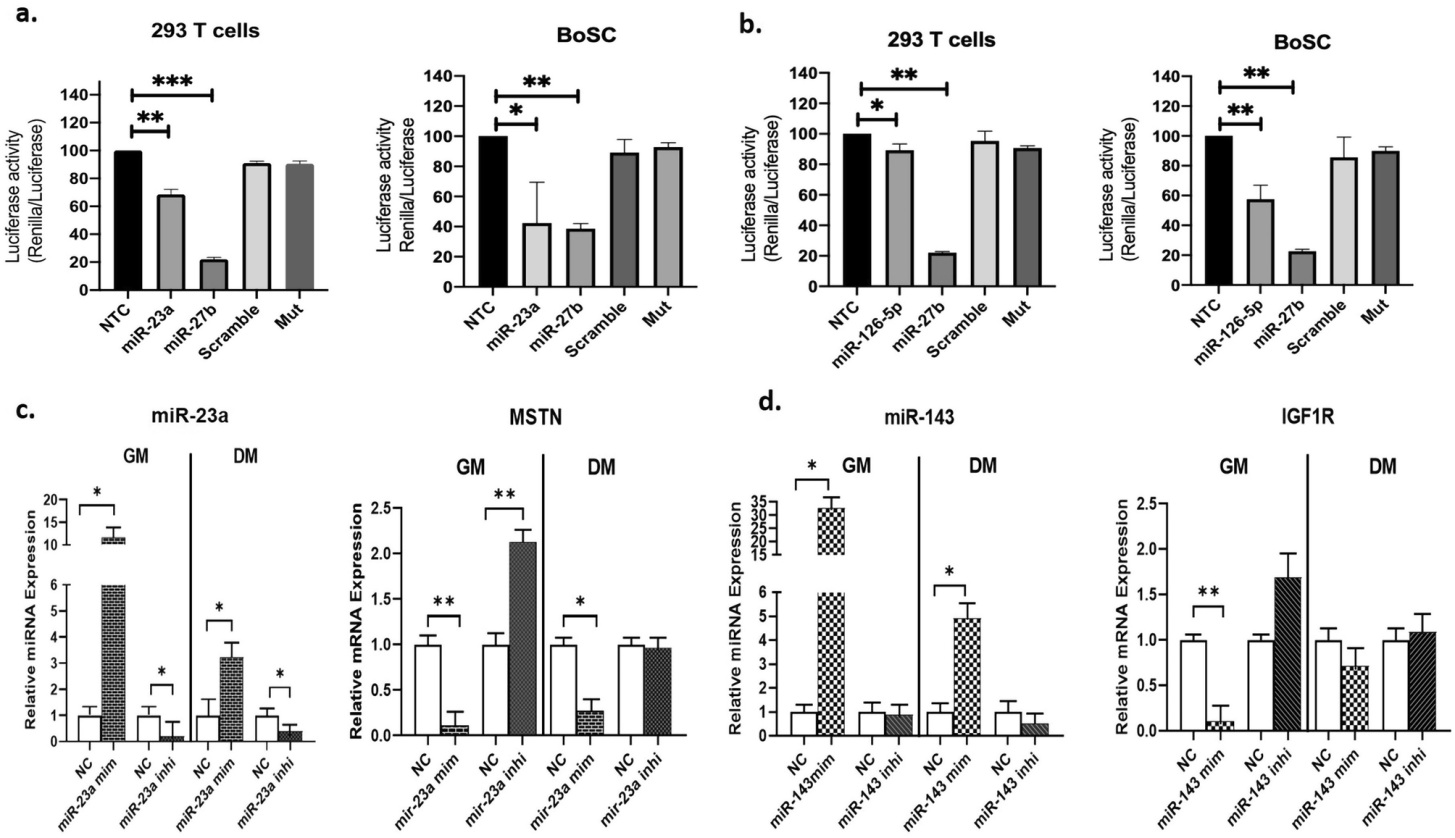
Luciferase assays and target gene expression analysis. 293T and BoSC were co-transfected with psi-check 2 reporter vectors containing native or mutated MSTN 3′UTR and miR-23a and miR-126-5p mimics followed by cell lysis and luciferase assay 24 h later. Statistical significance was calculated for p < 0.05. *NTC* non-treated cells, *Mut* MSTN 3′-UTR mutated. (**a**) Dual luciferase activity inhibition by miR-23a in 293T cells (34%, p < 0.05) and BoSC (57%, p < 0.05). (**b**) Dual luciferase activity inhibition by miR-126-5p in 293T cells (11%, p < 0.05) and BoS C (43%, p < 0.05). (**c**) Upregulation or downregulation of miR-23a levels affected the expression of MSTN gene expression in BoSC when transfected with miR-23a mimic, miR-23a inhibitor and compared with negative control (scramble). (**d**) Upregulation or downregulation of miR-143 levels affected the expression of IGF1R gene expression in proliferating myoblasts and differentiated myotubes when transfected with miR-143 mimic, miR-143 inhibitor and compared with negative control (scramble). Differences between the expression level of negative control and miR-143 mimic and inhibitor were assessed by Student *t* test. Differences in gene expression are shown as fold change (mean ± SEM). Significance expressed as *p < 0.05, ***p < 0.0005.

Among the 7 ci-miRNAs selected on the base of expression characteristics described above, TargetScan version 7.2 predicted MSTN as one of the target genes for miR-23a and miR-126-5p. To investigate the mechanism of miR‐23a and miR-126-5p, transfection of miRNAs mimic with luciferase reporter gene cloned to the wild-type 3′-UTR of MSTN and mutated MSTN 3′-UTR were performed in both 293T cells and myoblasts (BoSC in GM). miR-27b mimic and scramble were used as positive^15^ and negative controls, respectively (Fig. 5a,b). Both the miRNAs confirming the direct binding with the wild-type MSTN 3′-UTR. miR-23a significantly inhibited the dual luciferase activity around 34% and 57% in 293T and myoblasts respectively, and no inhibition of MSTN 3′-UTR-mutated was identified (Fig. 5a). Although miR-126-5p significantly inhibited in 293T cells and in myoblasts, the binding activity was lower in both the cell systems, 11% and 43% respectively, probably ascribable to the different context score attributed to TargetScan (Fig. 5b).

### miR-23a and miR-143 overexpression modulates skeletal muscle hypertrophy-related target genes

Several published results suggested that MSTN as well as Insulin Like Growth Factors (IGFs) were crucial mediators in physiological skeletal muscle processes, including development, hypertrophy, and regeneration^33–35^. Based on the above results related to luciferase activity inhibition, we decided to proceed investigating only the gain and the loss of expression of miR-23a on MSTN because it demonstrated a stronger direct binding compared with miR-126-5p. miR-143 was additionally investigated because TargetScan revealed a good predictive context score for its putative target gene IGF1R, previously demonstrated as functional target of this miRNA^36^.

To test the ability of miR-23a and miR-143 to alter the expression of their targets in myoblasts under GM and DM conditions, the overexpression of both miRNA mimics was induced and the post-transfection expression of miR-23a and miR-143 and their target genes MSTN and IGF1R respectively, at mRNA levels were defined through qRT-PCR (Fig. 5c,d).

The overexpression of miR‐23a (log2fold change = 11, p < 0.05) induced a significant decrease in MSTN expression in proliferative (GM, p < 0.004) and in differentiated (DM, p = 0.04) myoblasts (Fig. 5c). Conversely, knockdown of miR‐23a through miR-23a inhibitor (log2fold change = 0.02, p < 0.05) increased MSTN expression in proliferative myoblasts (p < 0.002), but not in differentiation condition (Fig. 5c). These results revealed that the overexpression of miR‐23a inhibited MSTN mRNA expression in myoblasts.

In proliferating myoblasts (GM), comparable results were obtained with the overexpression and the inhibition of miR-143 with significant different levels of IGF1R expression when compared with control after 24 h of transfection (Fig. 5d). Overexpression of miR-143 (log2fold change = 32, p < 0.05, Fig. 5d) significantly reduced the expression of IGF1R (Fig. 5d). In differentiated condition, no significant differences in IGF1R expression emerged as well as the inhibition of miR-143 showed no different significant expression in both the myoblasts culture conditions (Fig. 5d).

### Relationships between miRNAs expression and animals’ body weight

**Figure 6:**
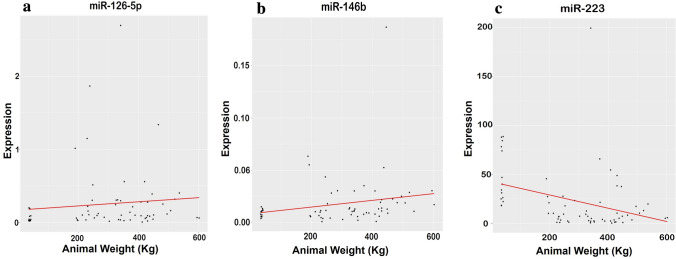
Plot of Spearman correlation results showing the correlation between animal weight (Kg) and miR-126-5p, miR-146b and miR-223 expression. (**a**) Shows the positive correlation between the expression of miR-126-5p and animal weight (kg) rho = 0.26 (p = 0.02) (**b**) shows the positive correlation between the expression of miR-146b and animal weight (kg) rho = 0.36 (p = 0.002) (**c**) shows the negative correlation between the expression of miR-146b and animal weight (Kg) rho = − 0.39 (p = 0.001). The solid line indicates the correlation.

To further deepen the relationship between the animal weight and the expression of all the 14 ci-miRNAs validated in qRT-PCR, we analysed the Spearman's rank correlation coefficients. Considering all the time points, the statistical test highlighted that miR-126-5p and miR-146b were positively correlated to animals' weight, rho = 0.26 (p = 0.02) and rho = 0.36 (p = 0.002) respectively (Fig. 6a,b), while a negative correlation was observed for miR-223 expression and beef cattle weights, rho = − 0.39 (p = 0.001) (Fig. 6c).

## Discussion

In the recent years, tissue and circulating miRNAs have gained a lot of attention due to their legit fine-tune role in orchestrating several physiological processes, metabolic changes and adaptive response pathways underlying skeletal muscle growth, differentiation, and hypertrophy^37^. However, mostly the studies have been carried out on tissue miRNAs in humans and rodent models. The knowledge about ci-miRNAs in livestock and particularly in bovine is limited. To characterize the changes in skeletal-muscle related ci-miRNAs during the growth of Piedmontese beef cattle, we examined the plasma samples of animals categorized into four groups based on the age and corresponding weight.

The goal of this study was to characterize the plasma miRNA signatures of cattle from first week of life until the time of commercial end of the fattening period before slaughter to integrate information between ci-miRNAs expression, muscle physiological age-growth, and animals’ weight. We adopted a multistep approach, using firstly a miRNAomics approach to identify DE-miRNAs in plasma, followed by validation of miRNAs profiling results through qRT-PCR. Furthermore, the investigation of ci-miRNAs along with gene targets involved in skeletal-muscle physiology mechanisms including hypertrophy was carried out demonstrating MSTN as new direct target of miR-23a (Fig. 4a) and miR-126-5p (Fig. 4b). At last, we found correlation between ci-miRNAs expression and animals body weight.

In our experiments, the levels of myomiRNAs including miR-1, miR-133a/b, and miR-206 were found negligible in all the age groups on sequencing. Muroya and colleagues reported a similar result obtained by microarray analysis done in the plasma of 22 months aged Japanese beef cattle^38^. Circulating levels of myomiRNAs are described increased in patients of muscular pathologies^39,40^, and their higher abundance in blood stream of patients with skeletal muscle pathologies suggest a possible relation with the injury of the fibres.

Among the DE-miRNAs identified by qRT-PCR, miR-10b, miR-126-5p, miR-143 (Fig. 3a), and miR-146b were found to be increased in expression along the age-groups when compared with NB and reached the highest expression at 10–12 months for the first three miRNAs, and at 15–17 months of life for miR-146b (Fig. 4a).

Previously, miR-10b, miR-143 and mir-146b were described as involved in PI3K/AKT pathway in the skeletal muscle tissue and myoblast cells^18,41,42^. It seems that all these miRNAs negatively regulated the myoblasts proliferation. A study reported that miR-10b significantly suppressed PIK3CA expression and decreased PI3K/Akt/mTOR pathway activity, promoting expression of TGF-β^43^. Furthermore, miR-10b expression steadily decreased during myoblasts proliferation, but significantly increased during myoblasts differentiation in C2C12 mouse cell line by binding with NFAT5 gene (isoform of Nuclear Factor of Activated T cells) which is recognized as a key regulator in myoblast migration and differentiation^44^.

Additionally, miR-10b was previously identified upregulated in grazing cattle plasma compared with grain-fed animals coincidently with the expression change of miR-10b in the *Longissimus dorsi.* This observation was correlated with possible alterations of skeletal muscle regulation through PTEN targeting (the phosphatase that converts phosphatidylinositol 3,4,5-triphosphat), involved in muscle cell differentiation and hypertrophy^45^.

Likewise, miR-143 was demonstrated to have a role in skeletal muscle and adipose tissues through the regulation of target genes involved in PI3K, IGF and FoxO signaling pathways such as IGF-I, IGF-II, IGF-IR, and IGFBPs^18,46^. The pathway analysis prediction (KEGG) of our sequencing data revealed a relation between the same pathways and ci-miRNAs identified in bovine plasma (Fig. 2).

In in vitro bovine cell models, miR-143 exerted important function in bovine myogenesis by targeting IGFBP5 (insulin like growth factor binding protein 5)^18^, which is an important component of IGF signalling pathway, but also its expression increased in differentiating bovine satellite cells^47^. At the end of pigs’ fattening period, miR-143 expression was found upregulated in the psoas muscle, assuming its involvement in processes for the transformation of muscle fibre type^19^, but an important positive role of miR-143 was already identified in adipogenesis mechanisms^48^ and recently demonstrated highly expressed in the adipose tissue of cows^49^.

In our findings, the increased levels of expression of circulating miR-10b and miR-143 during 10–12 months of age could be suggestive of their involvement in differentiation processes when the animal substantially gains the body weight, but the increase in miR-143 expression could be also linked with adipocyte differentiation. So, it can be hypothesized that this trend might mediate the maintenance of proliferating status of myoblasts during the first months of life and then induces the differentiation of muscle and adipose tissue at the end of the commercial fattening period. Indeed, according to Bonnet and colleagues, in cattle, the increase in number and differentiation of adipocytes at intramuscular level is described beginning from 12 months of age^3^.

In addition, we found miR-146b differentially expressed in the plasma when NB subjects and the elder groups were compared (Fig. 4a) and it was positively correlated with the body weight gain during the four growth points (Fig. 6b). In mouse and chicken models, miR-146b was described as a positive regulator of myogenic differentiation, acting through multiple targets (e.g., Smad4, Notch1, and AKT1)^20^ but this function was demonstrated in tissue. At present, no information is available about the role of plasma miR-143 and miR-146b in cattle, but their differential expression among ages in the bloodstream might attribute to an important role in the regulation of growth mechanisms probably related also to skeletal muscle^42^.

MSTN is a secreted growth and differentiation factor that belongs to TGF-β superfamily. MSTN gene mutation or targeted degradation of its mRNA has shown increased muscle mass attributing to muscle hypertrophy phenotype^15^.

In our qRT-PCR assay, miR-23a showed high expression in terms of Cq values in all the age-groups. Even if not differentially expressed, this result combined with the targeting of MSTN mRNA by miR-23a are suggestive of an important role of this miRNA in Piedmontese breed affecting the double-muscled phenotype. Our previous study has shown functional effect of miR-27b on MSTN and as one of possible contributing factors of hypertrophy in Piedmontese cattle^15^. Here we have demonstrated for the first time the direct binding between miR-23a and MSTN gene. Indeed, both in cardiac^50^ and in skeletal muscle^51^ it was previously demonstrated that miR-23a can mediate the hypertrophic signals, through the binding of an anti-hypertrophic protein (NFATc3) and two muscle‐specific ubiquitin ligases (MAFbx/atrogin 1 and MuRF1) that promote hypertrophy by protecting muscle atrophy‐associated protein degradation. These findings suggest that the high level of miR-23a expression in Piedmontese breed may have role in modulation of skeletal muscle mass. Future studies are required to specifically elucidate the role of miR-23a and if it can exert a synergic effect with other miRNAs such as miR-126-5p and miR-27b in the hypertrophic program.

Interestingly, miR-223 showed a decreased expression in the elder animals when compared with NB and its expression was negatively correlated with their weights. TargetScan prediction indicated IGF1R as a putative target of miR-223. Recent studies have described miR-223 as involved in various myocardial disorders related with cardiomyocytes hypertrophy also through the IGF1R binding^52,53^. It seems that the NB subjects with higher levels of miR-223 show higher body weight at 10–12M, further analysis will confirm with a larger sample number if miR-223 could be defined as predictive marker in differential growth.

## Conclusion

For the first time, our findings provide evidence of high numbers of skeletal-muscle related miRNAs in beef cattle plasma of different age-groups. Among these ci-miRNAs, miR23a and miR-126-5p were demonstrated to directly bind MSTN mRNA in a bovine satellite cells in vitro model. Three of DE ci-miRNAs (miR-146b, miR-126-5p and miR-223) have shown correlation between miRNAs expression value and body weight during different age. All these results are the basis for future investigations about a possible role of ci-miRNAs as biomarkers of muscle post-natal growth and development. Remarkably, the information about plasma miRNAs associated with production traits in beef cattle could prove to be promising biomarkers for the genetic selection of meat-purpose animals for future breeding. The pattern of miRNAs expression and its correlation with age growth status are, obviously, intriguing to predict at calf birth and the trend of weight during the first months of life.

## Material and methods

### Ethics statement

Blood samples were collected from Piedmontese cattle housed in the Animal Facility of Dept. of Veterinary Science, University of Turin, with the authorization of Ethical Animal Welfare Committee (Prot. No. 663).

### Blood collection and plasma separation

Blood samples were collected in 10 ml K2 EDTA Vacutainer tubes (Becton Dickinson, USA) by jugular vein puncture, using 18G needles (Becton Dickinson) and instantly stored in ice. Within 2 h of collection, samples were centrifuged at 1900*g* for 10 min at 4 °C to remove blood cells, followed by second centrifugation at 16,000*g* for 10 min at 4 °C to remove cellular debris and platelets^54^. One aliquot of 500 µl of each plasma sample was immediately used for RNA extraction and the remaining aliquots were frozen at − 80 °C. Blood samples were collected from ‘N’ no. of animals falling into four age groups: N = 15 samples/NB; N = 18 samples/4–6 months; N = 18 samples/10–12M; N = 16 samples/15–17M.

### RNA extraction and cDNA synthesis

Total RNA was extracted using Maxwell RSC miRNA Plasma and Serum kit (Promega, USA) according to the manufacturer’s protocol. Before RNA extraction from 500 µl of plasma sample, 1 µl of UniSp2, 4, 5 (miRCURY LNA spike-in, Qiagen, USA) was spiked-in as an internal control. A quantity of 0.5 µl of a mix of ce-miR39 and Unisp6 (miRCURY LNA spike-in, Qiagen, USA) was added as an internal control. Quantification of miRNA was performed by Quantus 3.0 fluorometer (Invitrogen, Life technologies, USA).

Reverse transcription (RT) was performed using miRCURY LNA RT II Kit (Qiagen, USA) according to manufacturer’s protocol. cDNA was synthesized from 0.8 µl of total RNA. A quantity of 0.5 µl of Unisp6 (miRCURY LNA spike-in, Qiagen, USA) was added as an internal control.

For the RNA extraction from cells, TRI Reagent (Sigma-Aldrich, St. Louis, MO, USA) was used following the manufacturer’s protocol. Cells were homogenized in 1 ml of TRI reagent and RNA pellets were resuspended in variable amount of RNAse free water corresponding to the size of pellet. RNA quantity and quality were determined using Nanodrop ND1000 Spectrophotometer (Thermo Fisher Scientific, USA). The ratio of the optical densities measured at 260 and 280 nm was > 1.9 for all the RNA samples. cDNA was synthesized using iScript Reverse Transcription Supermix for qRT-PCR kit (Bio-Rad, USA) for each sample taking the volume of eluted miRNAs sample equivalent to 500 ng.

### Small RNA sequencing: library preparation and sequencing

**‘**TruSeq SmallRNA Sample Prep kit’ (Illumina, San Diego, CA) was used for library preparation following the manufacturer’s instructions. RNA samples were previously quantified, and quality tested by Agilent 2100 Bioanalyzer RNA (Agilent technologies, Santa Clara, CA) or by Caliper RNA LabChip GX (Caliper Life Sciences, Hopkinton, MA). Final libraries were quantified using Qubit 2.0 Fluorometer (Invitrogen, Carlsbad, CA) and quality tested by Agilent 2100 Bioanalyzer (Agilent technologies, Santa Clara, CA) or by Caliper RNA LabChip GX (Caliper Life Sciences, Hopkinton, MA). Libraries were then prepared for sequencing and sequenced on single-end 75 bp mode on NextSeq500 (Illumina, San Diego, CA). The Bcl2Fastq 2.0.2 version of the Illumina pipeline was used to process raw data for both format conversion and de-multiplexing. Adapter sequences were masked with Cutadapt v1.11 from raw Fastq data using the following parameters: -anywhere (on both adapter sequences) -overlap 5 -times 2 -minimum-length 35 -mask-adapter. Lower quality bases and adapters were trimmed by ERNE v1.4.6 software. Above mentioned small RNA-Seq procedures were commercially performed by IGA Technology Services (IGAtech), Udine, Italy (Fig. 1b). GLM analysis was performed cumulatively for all the four groups using DESeq software taking into consideration log2 fold change > ± 0.58 and p < 0.05^55^.

### miRNAs pathway analysis and target gene prediction

Bovine miRNAs were related to their miRNA family through TargetScan 7.2 association table^56^. miRNA-target genes were associated to our miRNAs of interest by selecting from TargetScan 7.2 predicted target genes with at least one conserved binding site and a cumulative weighted context++ scores (CWCS) > − 0.05^57^ KEGG (Kyoto encyclopaedia of genes and genomes) enrichments and functional analysis were conducted through Bioconductor^58^ package ClusterProfiler, version 3.12.0. A 0.05 cut off value was chosen for both p value and q value and BH (Benjamini–Hochberg) and False Discovery Rate control method for multiple testing was considered. Human functional annotations were based on org.Hs.eg.db packages. All analyses were run in R, a free software environment for statistical computing and graphics, release 3.6.3^59,60^. 

### Quantitative assessment of miRNAs and mRNA target gene expression

qRT-PCR with total cDNA was performed using SYBR green II PCR Kit (Qiagen, Germany). PCR amplifications were performed on Bio-Rad CFX Connect Real-Time System (Bio-Rad, Hercules, CA, USA). The qRT-PCR parameters specific for miRNAs and gene expression are detailed in Supplementary Table S4. Differential expression among miRNAs was carried out by comparing the normalized Cq values (ΔCq) for all biological replicates between the two group of samples. Based on stable number of sequencing reads and stable value of qRT-PCR expression among the groups, miR-378 was selected as the reference gene. The fold change of expression of transcript/miRNA was calculated by 2^−ΔΔCq^ method where ΔCq = Cq of the target gene/miRNA − Cq of the reference gene/miRNA^12^. Data were expressed as fold-change with respect to new-born (NB) samples.

For the quantitative expression analysis of target genes, cDNA (5 μg) was prepared in a single run to perform qRT-PCR experiments for all the selected genes. To determine the relative amount of specific IGF1R and MSTN transcripts (Supplementary Table S3), the primers for target and reference genes were designed on Bos taurus GenBank mRNA sequences using Primer 3 Software (version 4.0). Oligonucleotides were designed to cross the exon/exon boundaries to minimize the amplification of contaminant genomic DNA and were analysed with the IDT tool^61^ for hairpin structure and dimers formation. Primer specificity was verified with BLAST analysis against the genomic NCBI database. To establish primers efficiency, the dilution method was used. Hypoxanthine phosphoribosyl transferase 1 (HPRT-1) gene was used as a reference gene for RNA concentration and reverse transcription efficiency^12^. Tables 1 and 2 summarize primer assays information including sequences and target gene assays with gene accession number and amplicon sizes. Primers were pre-designed and commercially synthesized by LNA technology (Qiagen, USA) (see Supplementary Table S2).

### Cell culture

Primary bovine satellite cells (BoSC) were isolated and cultured from bovine *longissimus dorsi* muscle, as previously reported^12^ and 293T cell line (ATCC, Rockville, MD, USA) was obtained from the American Type Culture Collection. 293T cells and BoSC were cultured in growth medium (GM) containing high‐glucose Dulbecco's modified Eagle's medium (DMEM) (Sigma-Aldrich, USA), supplemented with 1% penicillin‐streptomycin (Sigma-Aldrich, USA) and 10% fetal bovine serum (FBS) (Euroclone) and 20% FBS plus 10% Horse Serum (Sigma-Aldrich, USA) respectively. Cells were incubated at 37 °C in a 5% CO_2_ humidified atmosphere. The myoblasts differentiation was induced by replacing GM with differentiation medium (DM) (DMEM 2% horse serum) when BoSC confluence reached nearly 70–80%.

### Transfections and dual-luciferase reporter assay

293T cell line and BoSC were used to perform transfections for dual-luciferase assay. Cells were seeded at the count of 100,000 cells per well of a 24-well plate. At 60–70% confluence cells were transfected. A total of 300 ng of the appropriate psicheck-2 luciferase reporter construct cloned with 3′-UTR of the MSTN transcript^15^ was co-transfected with 30 nM final concentration of double-stranded RNA oligonucleotides designed to mimic miR-23a, miR-126-5p (Qiagen, Germany) and miR-27b (Exiqon, USA) molecules using Lipofectamine 2000 (Invitrogen, USA) according to the manufacturer’s protocol. Twenty-four hours after transfection, the medium was changed, and cells were grown for additional 24 h before assay. Firefly and Renilla luminescent signals were quantified according to the manufacturer’s instructions by Dual-Luciferase Reporter Assay System (Promega, USA) with a VICTOR Multilabel Counter luminometer (PerkinElmer, Waltham, MA). All values are given relative to transfections with the appropriate negative (Scramble) and positive (miR-27b) controls.

To study the effect of selected miRNAs on their target gene expression**,** BoSC were cultured in 6-well plates in GM and were transiently transfected with 30 nM of specific mimics and 100 nM of inhibitors of miR-23a and miR-143 (Qiagen, Germany) along with a negative control scramble (Exiqon, USA) using Lipofectamine 2000 (Invitrogen, USA). After 24 h, BoSC were harvested for the expression analysis of MSTN and IGF1R by qRT-PCR.

### Statistical analysis

DESeq software package was employed to perform the GLM analysis and pair-wise analysis on sequencing data. Statistical significance concerning qRT-PCR data was assessed on GraphPad Prism (9.0.2 version) by one-way ANOVA test and Student’s *t* test. Distribution normality assumption was assessed through Shapiro Wilk test. Data were expressed as mean ± SEM. Differences were considered as significant at a level of p < 0.05. Spearman's rank correlation coefficient was evaluated by cor.test function in R and used to test possible relationship between animal weight (expressed in Kg) and miRNA expression (https://www.r-project.org/). The level of significance has been set at p < 0.05 and trends were considered at 0.05 ≤ p < 0.1.

### Ethical approval and consent to participate

The cattle blood samples used in this scientific work were collected after the authorization of Ethical Animal Welfare Committee of Department of Veterinary Science, University of Turin (Prot. No. 663). All methods were carried out in accordance with relevant guidelines and regulations. Methods are reported in the manuscript following the recommendations in the ARRIVE guidelines.

## Supplementary Information

Supplementary Information.

## Data Availability

All data generated or analysed during this study are included in this published article [and its supplementary information files].

## Abbreviations

miRNAs: MicroRNAs
ci-miRNAs: Circulating microRNAs
MSTN: Myostatin
DE-miRNAs: Differentially expressed miRNAs
qRT-PCR: Quantitative real-time PCR
EDTA: Ethylenediamine tetra acetic acid
ERNE: Extended randomized numerical aligner
KEGG: Kyoto encyclopaedia of genes and genomes
HPRT-1: Hypoxanthine phosphoribosyl transferase 1
IGF1R: Insulin-like growth factor 1 receptor
BoSC: Primary bovine satellite cells
DMEM: Dulbecco's modified Eagle's medium
GM: Growth medium
DM: Differentiation medium
RPM: Reads per million
TGF-β: Transforming growth factor beta
PIK3CA: Phosphatidylinositol-4,5-bisphosphate 3-kinase catalytic subunit alpha
PI3K/Akt/mTOR: Phosphatidylinositol 3-kinase/alpha serine/threonine-protein kinase/mammalian target of rapamycin
PTEN: Phosphatase and tensin homolog
NFATc3: Nuclear factor of activated T-cells
MAFbx/atrogin 1: Muscle atrophy F-box gene
MuRF1: Muscle RING-finger protein-1
