# Supplementary material for: Circulating skeletal muscle related microRNAs profile in Piedmontese cattle during different age
Source: Sci Rep. 2021 Aug 4;11:15815. doi: 10.1038/s41598-021-95137-w (PMC8339070; doi:10.1038/s41598-021-95137-w)
Supplement: Supplementary file 1 — Supplementary Information. [file 41598_2021_95137_MOESM1_ESM.docx]

**Circulating skeletal muscle related microRNAs profile in Piedmontese cattle during different age**

**Rupal S Tewari^1^, Ugo Ala^1^, Paolo Accornero^1^, Mario Baratta^1^ and Silvia Miretti^1^***

^1^Department of Veterinary Science, University of Turin, Italy

*Corresponding author: Silvia Miretti (silvia.miretti@unito.it)

**Table S1:** The total number of reads per million obtained for each sample after removing reads with low quality (reads without adapter, short reads and reads with multiple undetermined base calls)

| **Library** | Total number of reads (*10^6) |
| --- | --- |
| NB_A1 | 11.18 |
| NB_A4 | 12.98 |
| NB_A6 | 12.59 |
| 04-06M_B3 | 16 |
| 04-06M_B4 | 14.88 |
| 04-06M_B5 | 6.85 |
| 10-12M_C1 | 12.23 |
| 10-12M_C2 | 11.25 |
| 10-12M_C3 | 9.7 |
| 15-17M_D2 | 4.75 |
| 15-17M_D4 | 7.03 |
| 15-17M_D5 | 4.7 |

**Figure S1:** Size distribution plot and read length distributions with a peak at 20–23 nucleotides which is the main feature of mature miRNAs.


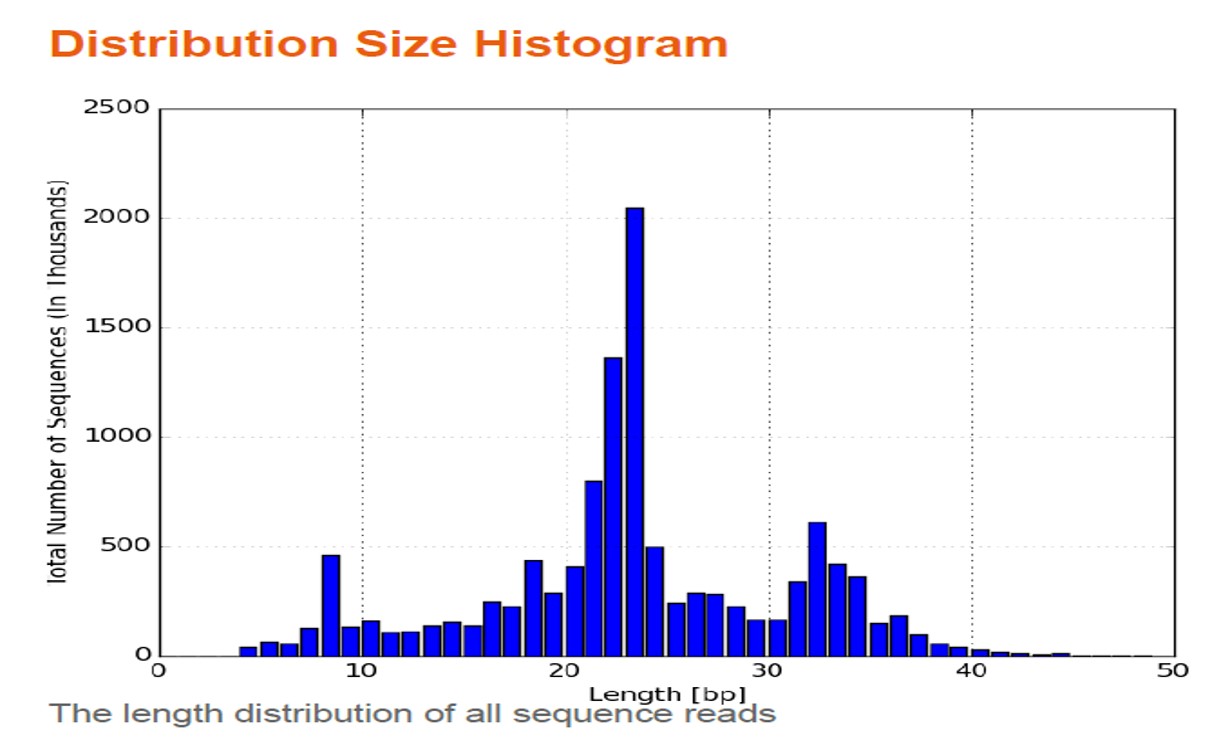


**Figure S2: Abundance of miRNAs in four groups.** No. of miRNAs were counted based on the expression level (RPM) for each group


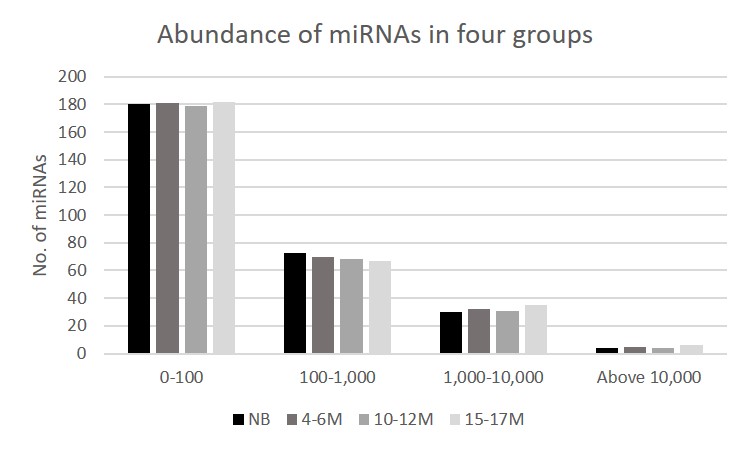


**Table S2:** **Primer assays used to detect bovine miRNAs expression through qRT-PCR**

| **miRNA** | **Primer sequence 5’-3’** |
| --- | --- |
| miR-126-5p | MIMAT0000444: 5'CAUUAUUACUUUUGGUACGCG |
| miR-10b | MIMAT0000254: 5'UACCCUGUAGAACCGAAUUUGUG |
| miR-143 | MIMAT0009233: 5'UGAGAUGAAGCACUGUAGCUCG |
| miR-223 | MIMAT0000280: 5'UGUCAGUUUGUCAAAUACCCCA |
| miR-30a-5p | MIMAT0003841: 5'UGUAAACAUCCUCGACUGGAAGCU |
| miR99a-5p | MIMAT0003537: 5'AACCCGUAGAUCCGAUCUUGU |
| miR-146b | MIMAT0005595: 5'UGAGAACUGAAUUCCAUAGGCUGU |
| miR-21-5p | MIMAT0003528: 5'UAGCUUAUCAGACUGAUGUUGACU |
| miR-221 | MIMAT0003529: 5'AGCUACAUUGUCUGCUGGGUUU |
| miR-30b-5p | MIMAT0000420: 5'UGUAAACAUCCUACACUCAGCU |
| miR-23a | MIMAT0003827: 5'AUCACAUUGCCAGGGAUUUCCA |
| miR-155-5p | MIMAT0000646: 5'UUAAUGCUAAUCGUGAUAGGGGU |
| miR-660 | MIMAT0004344: 5'UACCCAUUGCAUAUCGGAGCUG |
| miR-30c-5p | MIMAT0000244: 5'UGUAAACAUCCUACACUCUCAGC |

**Table S3: Primer assays for target genes**

| **Gene** | **Accession No.** | **5’ -3’ sequence** | **Amplicon size** |
| --- | --- | --- | --- |
| IGF1R | NM_001244612 | F: GGACGCAGTACGCCGTTTAC  R: AGGGAGGGCGGGTTCCACTT | 187 |
| MSTN | NM_001001525 | F: GGACGCAGTACGCCGTTTAC  R: AGGGAGGGCGGGTTCCACTT | 256 |
| HPRT1 | NM_0001942 | F: CGAGATGTGATGAAGGAGATGG  R: TGATGTAATCCAGCAGGTCAGC | 132 |

**Table S4: qRT-PCR parameters for miRNA assay and mRNA expression analysis**

| **Target to be quantified** | **qRT-PCR parameters** | **Kit used** |
| --- | --- | --- |
| **miRNA expression** | 1. 95°C for 15 mins 2. 94°C for 15 sec, slow ramp rate to 1.0C/sec 3. 56°C for 30 sec, slow ramp rate to 1.0 C/sec 4. 70°C for 30 secs, slow ramp rate to 1.0 C/sec   Repeat for 40 cycles  5. Melt curve 60°C to 95°C, increment of o.5C for 5 sec. | SYBR Green PCR Kit (Qiagen, Germany) compatible with cDNA made using miRCURY LNA RT II Kit (Qiagen, USA) |
| **Target gene expression** | 1. 94°C for 30 sec 2. 95°C for 10 sec 3. 60°C for 30 sec 4. 75°C for 5 sec 5. 95°C for 5 sec   Repeat steps 2, 3 and 4 for 39 cycles | Bio-Rad (Hercules, CA, USA) |
| **miRNA and gene expression in satellite cells** | 1. 95°C for 15 min 2. 94°C for 15 sec, slow ramp rate to 1.0 C/sec 3. 55°C for 30 sec, slow ramp rate to 1.0 C/sec 4. 70°C for 30 sec, slow ramp rate to 1.0 C/sec   Repeat all the steps for 40 cycles | SYBR Green PCR Kit (Qiagen, Germany) compatible with cDNA prepared with miSCRIPT II RT kit (Qiagen, Germany) |
